# Expert consensus on early orthodontic treatment of class III malocclusion

**DOI:** 10.1038/s41368-025-00357-9

**Published:** 2025-04-01

**Authors:** Xin Zhou, Si Chen, Chenchen Zhou, Zuolin Jin, Hong He, Yuxing Bai, Weiran Li, Jun Wang, Min Hu, Yang Cao, Yuehua Liu, Bin Yan, Jiejun Shi, Jie Guo, Zhihua Li, Wensheng Ma, Yi Liu, Huang Li, Yanqin Lu, Liling Ren, Rui Zou, Linyu Xu, Jiangtian Hu, Xiuping Wu, Shuxia Cui, Lulu Xu, Xudong Wang, Songsong Zhu, Li Hu, Qingming Tang, Jinlin Song, Bing Fang, Lili Chen

**Affiliations:** 1 Department of Stomatology, Union Hospital, Tongji Medical College, Huazhong University of Science and Technology, Wuhan, China; 2 School of Stomatology, Tongji Medical College, Huazhong University of Science and Technology, Wuhan, China; 3 Hubei Province Key Laboratory of Oral and Maxillofacial Development and Regeneration, Wuhan, China; 4 Department of Orthodontics, Peking University School and Hospital of Stomatology & National Center for Stomatology & National Clinical Research Center for Oral Diseases, Beijing, China; 5 State Key Laboratory of Oral Diseases & National Clinical Research Centre for Oral Diseases & Department of Pediatric Dentistry, West China Hospital of Stomatology, Sichuan University, Chengdu, China; 6 Department of Orthodontics, School of Stomatology, The Fourth Military Medical University, Xi’an, China; 7 State Key Laboratory of Oral & Maxillofacial Reconstruction and Regeneration, Key Laboratory of Oral Biomedicine Ministry of Education, Hubei Key Laboratory of Stomatology, School & Hospital of Stomatology, Wuhan University, Wuhan, China; 8 Department of Orthodontics, Beijing Stomatological Hospital, School of Stomatology, Capital Medical University, Beijing, China; 9 State Key Laboratory of Oral Diseases & National Center for Stomatology & National Clinical Research Center for Oral Diseases & Department of Orthodontics, West China Hospital of Stomatology, Sichuan University, Chengdu, China; 10 Department of Orthodontics, School and Hospital of Stomatology, Jilin University, Changchun, China; 11 Hospital of Stomatology, Guanghua School of Stomatology, Sun Yat-sen University, Guangdong Provincial Key Laboratory of Stomatology, Guangzhou, China; 12 Department of Orthodontics, Shanghai Stomatological Hospital & School of Stomatology, Fudan University, Shanghai, China; 13 Department of Orthodontics, The Affiliated Stomatological Hospital of Nanjing Medical University, Nanjing, China; 14 The Affiliated Hospital of Stomatology, School of Stomatology, Zhejiang University, Hangzhou, China; 15 Department of Orthodontics, School and Hospital of Stomatology, Shandong University, Jinan, China; 16 The Affiliated Stomatological Hospital, Jiangxi Medical College, Nanchang University, Nanchang, China; 17 Department of Orthodontics, Tianjin Stomatological Hospital, School of Medicine, Nankai University, Tianjin, China; 18 Department of Orthodontics, School and Hospital of Stomatology, China Medical University, Shenyang, China; 19 Nanjing Stomatological Hospital, Affiliated Hospital of Medical School, Nanjing University, Nanjing, China; 20 Xiangya Stomatological Hospital and Xiangya School of Stomatology, Central South University, Changsha, China; 21 School of Stomatology, Lanzhou University, Lanzhou, China; 22 Hospital of Stomatology, Xi’an Jiaotong University, Xi’an, China; 23 Hospital of Stomatology, Fujian Medical University, Fuzhou, China; 24 School/Hospital of Stomatology, Kunming Medical University, Kunming, China; 25 Shanxi Medical University School and Hospital of Stomatology, Taiyuan, China; 26 School of Stomatology, Zhengzhou University, Zhengzhou, China; 27 The First Medical Center, Chinese PLA General Hospital, Beijing, China; 28 Department of Oral and Cranio-Maxillofacial Surgery, Shanghai Ninth People’s Hospital, Shanghai Jiao Tong University School of Medicine & College of Stomatology, Shanghai Jiao Tong University, Shanghai, China; 29 State Key Laboratory of Oral Diseases & National Clinical Research Center for Oral Diseases & West China Hospital of Stomatology, Sichuan University, Chengdu, China; 30 College of Stomatology, Chongqing Medical University & Chongqing Key Laboratory of Oral Diseases & Chongqing Municipal Key Laboratory of Oral Biomedical Engineering of Higher Education, Chongqing, China; 31 Department of Orthodontics, Shanghai Ninth People’s Hospital, Shanghai Jiao Tong University School of Medicine & College of Stomatology, Shanghai Jiao Tong University & National Center for Stomatology & National Clinical Research Center for Oral Diseases & Shanghai Key Laboratory of Stomatology, Shanghai, China

**Keywords:** Bone development, Oral diseases

## Abstract

The prevalence of Class III malocclusion varies among different countries and regions. The populations from Southeast Asian countries (Chinese and Malaysian) showed the highest prevalence rate of 15.8%, which can seriously affect oral function, facial appearance, and mental health. As anterior crossbite tends to worsen with growth, early orthodontic treatment can harness growth potential to normalize maxillofacial development or reduce skeletal malformation severity, thereby reducing the difficulty and shortening the treatment cycle of later-stage treatment. This is beneficial for the physical and mental growth of children. Therefore, early orthodontic treatment for Class III malocclusion is particularly important. Determining the optimal timing for early orthodontic treatment requires a comprehensive assessment of clinical manifestations, dental age, and skeletal age, and can lead to better results with less effort. Currently, standardized treatment guidelines for early orthodontic treatment of Class III malocclusion are lacking. This review provides a comprehensive summary of the etiology, clinical manifestations, classification, and early orthodontic techniques for Class III malocclusion, along with systematic discussions on selecting early treatment plans. The purpose of this expert consensus is to standardize clinical practices and improve the treatment outcomes of Class III malocclusion through early orthodontic treatment.

## Introduction

Class III malocclusion is a type of malformation characterized by mesial molar relationship,^1,2^ often accompanied by anterior crossbite as the main feature. It is a developmental deformity resulting from a combination of genetic and environmental factors during childhood growth and development. The prevalence of Class III malocclusion varies in different regions. The prevalence ranges from 0.84% to 2.68% in America, 0.59% to 4.6% in Africa, 1.21% to 2.75% in Europe, and 3.91% to 6.46% in Asia.^1^ The prevalence of angle class III malocclusion varies greatly among and within populations. A study excluding children under 11 years old found that the populations from Southeast Asian countries (Chinese and Malaysian) showed the highest prevalence rate of 15.8%.^3^

Early intervention in severe skeletal Class III malocclusion is crucial for normal oral and maxillofacial development, facial esthetics, and healthy psychological development. It also simplifies treatment methods and shortens treatment duration. Timely intervention can lead to even better results. Therefore, the early treatment of Class III malocclusion is a significant area of expertise in orthodontics and a crucial task for orthodontists. Additionally, effective communication with parents to ensure their understanding and support, as well as fostering the child’s cooperation throughout the treatment process, are crucial components that can significantly impact the overall success of the treatment. The unpredictable nature of the malocclusion’s progression and the potential variability in treatment outcomes should be fully taken into account in early treatment of Class III malocclusion. Given the absence of standardized treatment guidelines for early correction of Class III malocclusion, establishing expert consensus is essential for guiding orthodontists.

## Methods

There are 33 experts who were solicited in this paper. Identifying the appropriate experts is critical to the Delphi process. The criteria for expert selection were: a high level of expertise in orthodontic evaluation, management and/or research, a willingness to complete digital surveys and/or attend consensus meetings, adequate level of written and spoken English, and/or peer-reviewed publication in orthodontic research. If possible specialists (1) had insufficient experience in assessing or managing orthodontics, and (2) had insufficient time to fully complete the online survey, they were excluded.

### Modified Delphi process

The study consisted of three rounds of purposeful numerical surveys. The literature was searched, the evidence was discussed, and a review of the evidence was completed by team members.

The first round consisted of a digital survey that posed open-ended questions to orthodontists and researchers with Class III malocclusion expertise. The initial round of surveys included open-ended qualitative information-gathering questions. The surveys for this study followed the CHERRIES^4^ and the reporting standard for conducting and reporting Delphi studies (CREDES)^5^ to avoid bias. The issues were outlined and presented for discussion. All group members who completed the survey were invited to participate in an online discussion session. Follow the nominal group consensus model and adopt a convenient, structured approach to gather qualitative information from the group.^6^ This approach has also been adopted for other consensus projects.^7,8^ During the discussion, the facilitators were impartial and ensured that the discussions were balanced to avoid “eminence bias“.^9^ They aimed to reach agreement, not to force consensus. After discussion, the key consensus statements were integrated and improved. Through the process of facilitating debate, the statements were gradually refined until the entire group was satisfied. The final round of Delphi involved further online surveys to test these statements by experts who met the previous inclusion/exclusion criteria. These are reviewed and revised by all the experts, and then the final version is received consensus from the experts.

## Etiology of class III malocclusion

### Genetic factors

Class III malocclusion, particularly anterior crossbite, has a significant familial tendency.^2,10–15^ However, simply inquiring about family history cannot differentiate the type of crossbite or predict the prognosis. As a polygenic inherited disease, anterior crossbite is influenced by both genetics and the environment. For patients with a family history of skeletal deformities, analyzing their dental and skeletal types, along with the family history, provides valuable insights. Some chromosomal disorders and single-gene genetic syndromes can affect jaw and tooth development, with anterior crossbite being one of the manifestations of these syndromes. These include Down syndrome (21-trisomy syndrome), craniofacial-clavicular dysplasia syndrome, Crouzon syndrome, and Rieger’s syndrome (Iris-dentition dysplasia syndrome),^16,17^ etc.

### Environmental factors

#### Congenital factors

Congenital cleft lip and palate is one of the significant causes of anterior crossbite.^18,19^ Due to the impact of cleft lip and palate on bone growth, as well as the limiting effect of surgical scars on maxillary development, cleft-related malocclusions often involve anterior crossbite or full-arch crossbite caused by underdevelopment of the maxilla. The incidence, location, and severity of crossbite are related to the type of cleft lip and palate. Generally, the more severe bone defects, the higher the incidence of crossbite and the greater likelihood of involving both sides of the arch.^19^ Congenital missing permanent upper teeth can also often be accompanied by anterior crossbite.

#### Acquired factors

##### Systemic diseases

Hyperfunction of the pituitary gland leading to excessive growth hormone production can cause conditions such as acromegaly, mandibular protrusion, and anterior or full dentition crossbite, particularly if it occurs after epiphyseal plate fusion.^20^ Rickets, due to vitamin D deficiency affecting calcium and phosphorus metabolism leading to bone metabolism disorders, can manifest as anterior crossbite and open bite due to mandibular developmental malformation.^21^

##### Respiratory diseases

Chronic tonsillitis can lead to tonsil hyperplasia and enlargement. To maintain respiratory tract patency and reduce compression stimuli, the tongue often protrudes forward and pulls the mandible forward, resulting in anterior crossbites and mandibular protrusion.^22–24^

##### Local obstacles in primary dentition and mixed dentition

Dental caries in primary teeth and the local obstacles in the primary dentition and mixed dentition caused by it are important acquired factors in the formation of anterior crossbite. The proximal caries of deciduous molars reduce the coronal width of the tooth and lead to a change in the tooth position, which then causes early contact and interference. These conditions are easy to trigger mandibular occlusion with forward or anterolateral path movements, forming an anterior crossbite. The early loss of primary teeth has a great impact on the development of occlusion. When most of the primary molars are lost prematurely, due to forced use of anterior teeth for chewing, the mandible gradually shifts forward, leading to anterior crossbite. The retained maxillary primary incisors force the permanent incisors to erupt on the palatal side, which creates a crossbite relationship with the opposing teeth. Insufficient wear of the deciduous canines can lead to early contact of the opposing canines, resulting in anterior crossbite or anterior and unilateral posterior crossbite.

##### Detrimental oral behaviors

protruding tongue, sucking fingers, biting the upper lip, mandibular protrusion habits, and incorrect artificial feeding can all cause anterior crossbite and mandibular protrusion.^23^ These bad habits will exert pressure on the teeth and jaw bones, change the coordination of the maxillofacial muscles, and eventually lead to malocclusion. Prolonged bad habits can increase the likelihood and severity of malocclusion.

### Epigenetic factors

Many studies showed that in addition to DNA sequence variation, some changes in gene expression may also be involved in the etiology of mandibular prognathism. Epigenetic mechanisms, such as DNA methylation and histone acetylation, regulate the transcriptional process without interfering with nucleotide sequence. The strength of masticatory muscles affects the development of the mandible and the maxilla, and the changes of the dimensions of the craniofacial skeleton affect the tension of masticatory muscles, leading to a suggestion regarding the epigenetic mechanisms involved in this bidirectional relationship. According to this theory, some environmental factors, such as forces acting on the mandible, may have an impact on epigenetic mechanisms, which regulates the expression of genes whose products are involved in mandible growth.^25^ Advances in high-throughput assessment technology and computational methodologies could lead to a better understanding of how the interaction of multiple genetic changes affects the onset and severity of Class III malocclusion. Since genes that increase susceptibility to skeletal Class III malocclusion have been identified in genome-wide association studies (GWAS), knowing the functions of the genetic loci systems’ genetics could most likely have the ability to understand the molecular basis and severity of the illness.^26^ Independent analysis on a patient population shows that expressions of both K(lysine) acetyltransferase 6B (KAT6B) and histone deacetylase 4 (HDAC4) are significantly greater in those with skeletal Class III malocclusion than in Class II malocclusion. Recent findings in craniofacial syndromes highlight the important role of KAT6B in epigenetic regulation of skeletal growth. It is a potent activator of RUNX2, which in turn activates osteoblasts and chondroblasts especially at the condylar level during normal growth and during bone regeneration after distraction osteogenesis. Epigenetic regulation through coordinate activities of both HDAC4 and KAT6B might be important in the entire masticatory musculoskeletal complex during the malocclusion development.^27^

## Clinical manifestations of class III malocclusion

### Abnormal dental relationship

Most of Class III malocclusion are manifested as anterior crossbite, which mostly involves six upper anterior teeth, and sometimes four incisors only. Dental anterior crossbite is characterized by lingual tipping of the upper anterior teeth and labial tipping of lower anterior teeth. The skeletal anterior crossbite is the opposite, showing that the upper anterior teeth are labially inclined and the lower anterior teeth are lingually inclined to compensate for the skeletal mal-position. The molar relationship is in a mesial position, with the mesially positioned mandible and mandibular dental arch. If the mandible moves forward by 1/4 of a molar or half the width of a premolar distance, where the mesial buccal cusp of the upper first permanent molar occludes to the distal buccal cusp of the lower first permanent molar, it is called a half-unit Class III malocclusion. If the mandible or mandibular dental arch is in an even more mesial position, where the mesial buccal cusp of the maxillary first permanent molar bites between the first and second permanent molars of the mandible, it is called a full-unit mesial malocclusion relationship. The length and width of the mandibular dental arch are larger than those of the maxillary dental arch, especially in the length. The maxillary anterior teeth often have varying degrees of crowding, while the mandibular anterior teeth have less crowding, even if there is a degree, it is relatively mild.

### Abnormal mandibular development and craniofacial relationship

#### Mandible

Excessive growth of the mandible leads to increases in both the total length of the mandible and specifically the length of its body compared to normal. The overall position of the mandible moves forward, with the joint, ascending ramus, mandibular angle, and chin all being in front. This often accompanies mandibular asymmetry and facial deviation.

#### Maxilla and midface

Insufficient forward development of the maxilla results in a decrease in maxillary length and a posterior position, causing a concave appearance in the midface.

#### Relationship between upper and lower jaws

Abnormalities often manifest as Class III skeletal facial type. The posterior cranial base is tilted downward and forward relative to the anterior cranial base, and an abnormal position of the cranial base promotes protrusion of the mandible. Bony mandibular protrusion often occurs with bony mandibular deviation.^28–32^

### Abnormal function of oral and maxillofacial system

#### Decreased chewing efficiency

Patients with anterior crossbite have an uncoordinated chewing activity. According to relevant research results, the chewing efficiency of patients with anterior crossbite is about half that of individuals with normal occlusion. Additionally, patients with this condition chew more times and for a longer time before swallowing food compared to normal individuals.^33^

#### Temporomandibular joint disorder

Anterior crossbite combined with temporomandibular joint disorder are not common. Although some patients show anterior condyle displacement on joint X-rays, clinical symptoms are not obvious. It is worth noting that in patients with mandibular protrusion but without anterior crossbite, the shallow overjet limits the tendency for mandibular forward development. This may lead to posterior displacement of condylar position and lead to temporomandibular joint disorder.^34^

#### Articulation and phonation problems

Anterior crossbite may affect speech, such as affecting the pronunciation of “s” and “z”. Anterior crossbite of Class III malocclusion may also be combined with posterior crossbite, which may lead to unstable tongue position during pronunciation and thus affect speech.^35^

#### Tooth wear and periodontal disease

Crossbite can lead to abnormal wear between incisors, which may result in pulpitis, occlusal trauma, and an increased risk of periodontal disease.

## Classification of class III malocclusion

### Classification by pathogenic mechanism

#### Dental type

Due to the obstacles in tooth eruption and replacement process, the abnormal position of the upper and lower incisors results in simple anterior crossbite.^36^ This anterior crossbite is of skeletal Class I (0° ≤ ANB angle ≤ 5°), and the molar relationship is mostly a mild mesial relationship. The maxillary anterior teeth are lingually inclined, and the mandibular anterior teeth are labially inclined. The correction is generally easy, with a good prognosis.

#### Skeletal type

The abnormal inter-jaw relationship caused by unbalanced growth of the upper and lower jaws,^36^ often manifests as excessive mandibular development, insufficient or normal maxillary development, skeletal Class III (ANB < 0°), and inability of the mandible to retract. The molar relationship is mostly a complete mesial relationship. The maxillary anterior teeth are labially inclined, and the mandibular anterior teeth are lingually inclined. Skeletal Class III, also known as true Class III malocclusion, is relatively difficult to treat and the severe cases may require orthognathic surgery.

#### Functional type

**Fig. 1:**
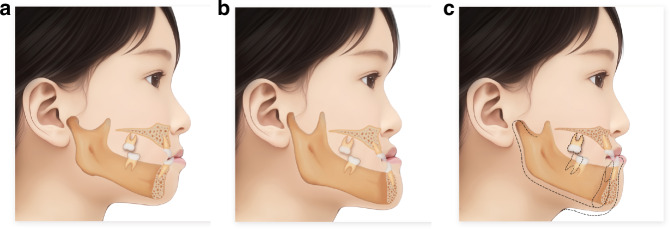
Schematic diagram of classification of class III malocclusion by pathogenic mechanism. **a** Dental type. **b** Skeletal type. **c** Functional type (The typical feature of functional Class III malocclusion is that the mandible can be retracted to the anterior teeth edge-to-edge position)

Any acquired crossbite involving neural-muscular participation and anterior displacement of the mandible, is called functional Class III malocclusion or pseudo-Class III malocclusion. The associated mandibular protrusion is called functional or pseudo-mandibular protrusion.^36^ Occlusal interference and early contact are the main causes of functional Class III malocclusion. Additionally, anterior crossbite and mandibular protrusion caused by bad oral habits, tonsil hypertrophy, etc., also belong to this type of functional malocclusion. In functional Class III malocclusion, the molar relationship is mostly mildly mesial, with generally small reverse overjet and deep reverse overbite. The size and shape of the mandible are basically normal, but the position is anteriorly shifted, showing slight mandibular protrusion and Class III facial type. The typical feature of functional Class III malocclusion is that the mandible can be retracted to the anterior teeth edge-to-edge position. When the mandible is retracted or in a resting position, the ANB angle increases significantly, and the profile improves significantly compared to the intercuspal position. Simple functional Class III malocclusion has a good treatment response and prognosis. Patients with functional Class III malocclusion often have varying degrees of skeletal abnormalities, and skeletal Class III cases may also have some functional factors. Due to the frequent coexistence of skeletal and functional factors, it is often difficult to definitively distinguish between functional and skeletal Class III malocclusion (Fig. 1).

### Classification by jaw characteristics

#### Sagittal types

The sagittal relationship of upper and lower jaws in Class III malocclusion can be divided into the following six types:^37^ normal maxilla with protruded mandible, retrognathic maxilla with normal mandible, normal maxilla with normal mandible, retrognathic maxilla with protruded mandible, protruded maxilla with protruded mandible, and retrognathic maxilla with retrognathic mandible.

#### Vertical types

**Fig. 2:**
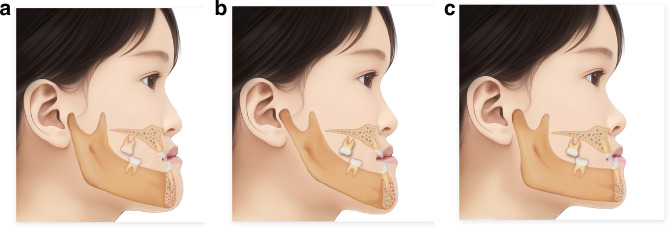
Schematic diagram of classification of class III malocclusion vertical types. **a** average angle type. **b** high angle type. **c** low angle type

The vertical bone facial type of Class III malocclusion can be divided into average angle type, high angle type, and low angle type.^37^ The mandibular plane angle of functional Class III malocclusion is generally flat, being low angle or average angle type; while in skeletal Class III malocclusion, the mandibular plane angle is steep, being high angle or average angle type (Fig. 2).

## Technology for early correction of class III malocclusion

Common orthodontic appliances used for Class III malocclusion include simple removable appliances, functional appliances, reverse-pull headgear, fixed appliances, and clear aligners (Table 1). Removable appliances, functional appliances and reverse-pull headgear are mainly used in the primary and mixed dentition periods; fixed appliances can be used for the early treatment of permanent teeth, among which “transmission straight-wire technique” can be selected as the first choice for the treatment of mild to moderate skeletal Class III malocclusion. The clear aligners can also be used for the treatment of primary teeth, mixed dentition and early permanent teeth according to the treatment needs.

**Table 1:** Comparison of technologies for early correction of class III malocclusion

Appliance	Remo-vable	Force-bearing part	Method	Indications	Contra-indications	Course of treat-ment
Removable appliance incorporating z-spring and posterior bite plate	yes	maxillary incisors	a posterior acrylic bite block to open the anterior reverse deep bite and z-springs to exert force to push the upper anterior teeth forward to eliminate anterior crossbite	anterior crossbite with moderate overbite resulted from lingual inclined upper anterior teeth in primary and mixed dentition phases	open bite	3–6 months
Lower Inclined Bite Plane (Catlan’s Appliance)	yes	maxillary incisor	the resin guide slope contacts the palatal side of the upper anterior teeth and guides the maxillary incisors to move forward	anterior crossbite cases with deep overbite, small overjet, and insufficient posterior teeth eruption in primary dentition	shallow overbite or big overjet	1–3 months
Fränkel III appliance	yes	Maxilla	remove the abnormal muscle forces in the labial and buccal areas that restrict the maxillary growth	during late primary to early mixed dentition stages to correct functional Class III malocclusion and skeletal Class III malocclusion characterized by maxillary retrusion and no mandibular prognathism	severe skeletal Class III malo-cclusion	1 year
Tooth-borne reverse-pull headgear	yes	maxilla and upper dentition	As the forward traction device uses the forehead and chin as anchorage sites, it promotes forward growth of both the maxilla and upper dentition while causing downward and backward rotation of mandible	patients with average or low mandibular angle	patients with high mandibular angle	1 year
Bone-borne reverse-pull headgear	yes	Maxilla	the titanium plate can be fixed on the maxilla as an intraoral device for anterior protraction	Class III malocclusion with obvious maxillary underdevelopment.	patients with poor general condition, poor blood coagulation function, intolerance to implant surgery, and poor bone quality	1 year
Maxillary protraction with inter-maxillary elastics to miniplates	no	Maxilla and mandible	Titanium plate implanted in jawbone to perform continuous intermaxillary traction	Class III malocclusion with obvious maxillary underdevelopment	poor general conditions, poor coagulation function, inability to tolerate implant surgery and poor bone quality	9–14 months
“2 × 4” Orthodontic Appliance	no	teeth	using the first permanent molar (or second deciduous molar) to provide anchorage for the initial correction of anterior crossbite	mixed dentition patients with dental anterior crossbite, especially for those with normal or lingual-tipping upper incisors and labial-tipping lower incisors with spacing and rotated anterior teeth	The anterior teeth root are flare-shaped.	6 months
Conventional Straight-Wire Appliance	no	teeth	Utilize the pre-set brackets to align the dentition, correct the anterior crossbite, and establish a neutral molar relationship	dental/functional and mild to moderate skeletal Class III malocclusions	severe skeletal Class III malo-cclusions	6–12 months
Transmission Straight-wire Therapy	no	teeth	Firstly, fine wires and light force are used together with Class III elastics to quickly correct anterior crossbite Then, rectangular wires are used to express the pre-set data of the bracket to achieve precise teeth control	dental/functional and mild to moderate skeletal Class III malocclusions	severe skeletal Class III malo-cclusions	3–6 months
Clear Aligners	yes	teeth	new orthodontic appliance characterized by 3D design and manufacture	patients with high esthetic requirements	Patients with poor compliance, severe skeletal Class III malo-cclusions	1 year
Lingual Orthodontic Appliance	no	teeth	The brackets are bonded to the lingual side of the teeth to perform treatment	patients with high esthetic requirements	severe skeletal Class III malo-cclusions	1 year

### Removable appliance incorporating Z-spring and posterior bite plate

**Fig. 3:**
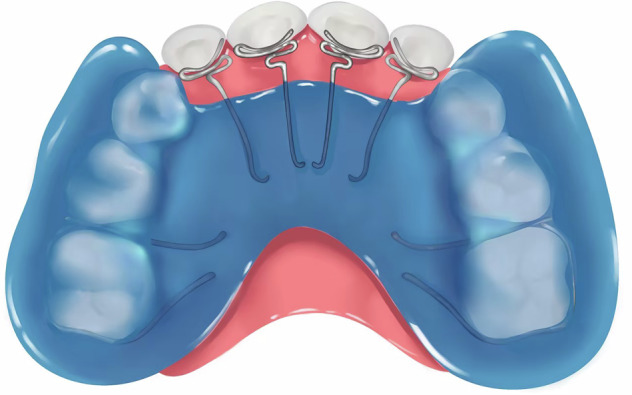
Schematic diagram of removable appliance incorporating z-spring and posterior bite plate

A simple removable appliance (Fig.3) designed with a posterior acrylic bite plate to open the anterior reverse deep bite and z-springs to exert force to push the upper anterior teeth forward to eliminate anterior crossbite. The plane of the spring should be perpendicular to the long axis of the maxillary incisors. After the anterior crossbite is resolved and normal overbite and overjet are established, the posterior bite plate can be decreased gradually (recommended 0.5-1 mm in thickness each time until fully removed). For this type of appliance, revisits for adjustment are usually every 2-3 weeks, with the lingual z-springs opened by 1 mm each time, and it should be worn all day. This appliance is suitable for anterior crossbite with moderate overbite resulting from lingual inclined upper anterior teeth in primary and mixed dentition phases. It is not suitable for patients with a tendency for open bite. The treatment duration for anterior crossbite correction with this type of appliance is generally 3–6 months.

### Lower inclined bite plane (Catlan’s appliance)

**Fig. 4:**
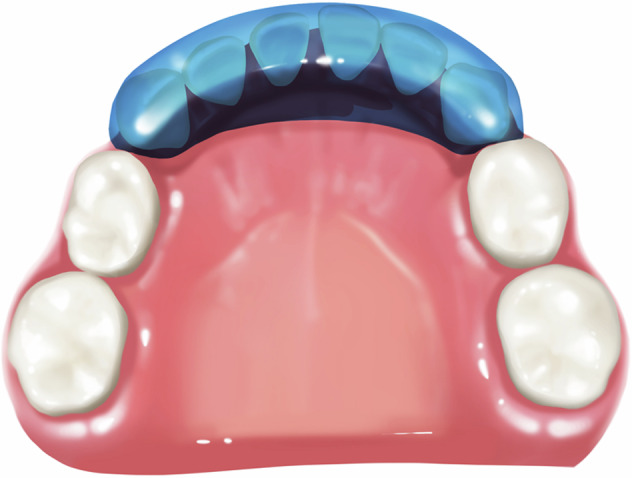
Schematic diagram of lower inclined bite plane

This inclined plane (Fig. 4) with a slope of 45° to the long axis of the tooth is created using acrylic material. The acrylic inclined plane is cemented onto the mandibular incisors and canines using dental cement (zinc oxide eugenol cement). After cementation, the resin guide slope contacts the palatal side of the upper anterior teeth and guides the maxillary incisors to move forward to eliminate anterior crossbite. Patients are suggested to adopt a softer diet than usual for the first few days. They need to pay more attention to keep good oral hygiene to avoid gingivitis and are recalled every 2-week to clinically evaluate the treatment progress. It is suitable for anterior crossbite cases with deep overbite, small overjet, and insufficient posterior teeth eruption in primary dentition. The treatment duration for anterior crossbite correction with this type of appliance is generally 1–3 months.

### Fränkel III appliance

**Fig. 5:**
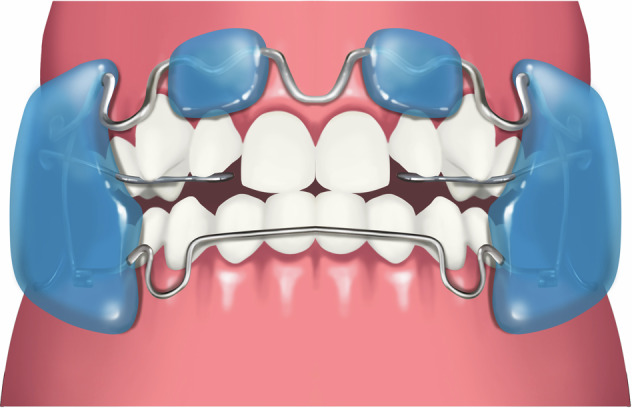
Schematic diagram of Fränkel III appliance

Fränkel III (Fig. 5) is a functional regulator appliance developed by Rolf Fränkel, Germany in 1966. This appliance is used to remove the abnormal muscle forces in the labial and buccal areas that restrict the maxillary growth, thereby, providing an environment which maximizes skeletal growth of the maxilla and eliminates anterior and posterior crossbites. The restricting effect of the buccinator and orbicularis oris muscles on maxillary skeletal development is prevented by means of lip pads and vestibular shields, thus maxillary development is stimulated. This appliance is used to correct functional Class III malocclusion and mild skeletal Class III malocclusion characterized by maxillary retrusion and no mandibular prognathism. The treatment duration for using Fränkel III appliance for maxillary growth modification is generally 1 year.

### Tooth- and/or mucosa-borne reverse-pull headgear

**Fig. 6:**
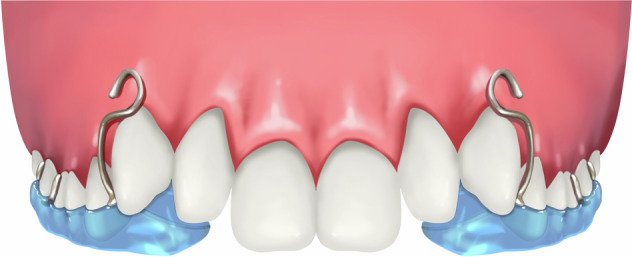
Schematic diagram of tooth-borne reverse-pull headgear

It is an orthopedic orthodontic appliance (Fig. 6) primarily aimed at promoting maxillary bone development. It is bonded to the dentition through Hyrax or Hass expander with welded traction hooks extended to the canine area. Elastics are used to connect the reverse-pull headgear with the intra-oral appliance to protract the maxilla in a forward and downward direction. Usually, a rapid maxillary expansion is first performed for 3-4 weeks,^38,39^ which helps to loosen the maxillary sutures, followed by protracting the maxilla to promote new bone deposition at the maxillary sutures while stretching the periosteum of the maxilla and promoting forward growth of the maxilla and upper dentition.^40,41^ As the tooth-borne reverse-pull headgear uses the forehead and chin as anchorage sites, it promotes forward growth of both the maxilla and upper dentition while causing downward and backward rotation of the mandible.^39^ For patients with average or low mandibular angle, clockwise rotation of the mandible is beneficial to improve the profile; however, for patients with high mandibular angle, downward and backward rotation of the mandible will further increase the lower facial height and result in unesthetic profile. Therefore, reverse-pull headgear should be cautiously used in high-angle Class III patients. Generally, the protraction force is between 350 and 500 g on each side, worn for more than 13–16 h a day.^42^ The treatment duration for using this appliance combined with expansion for maxillary growth modification is generally 1 year.

### Bone-borne reverse-pull headgear

**Fig. 7:**
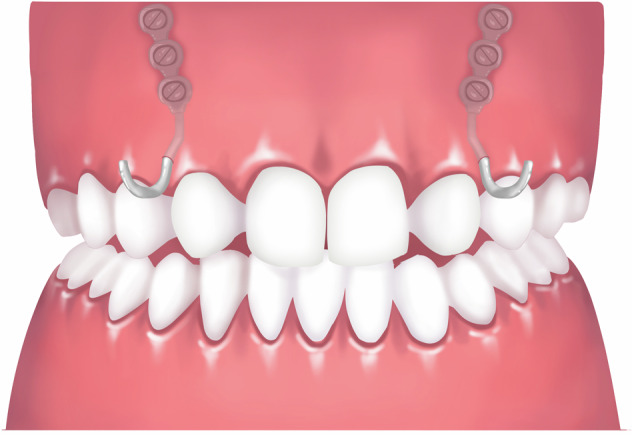
Schematic diagram of bone-borne reverse-pull headgear

Traditional tooth- and/or mucosa-borne reverse-pull headgear generally results in maxillary skeletal protraction but is frequently accompanied by unfavorable dentoalveolar effects, such as upper incisor protrusion and dental crowding aggravation.^43,44^ To achieve the best skeletal effect, the titanium plate can be fixed on the maxilla as an intraoral device for anterior protraction, achieving better bone modification effect.^45–47^ The titanium plate could be 3D customized based on the patient’s CBCT.^48^ To avoid damage to the tooth root, a single titanium plate can be implanted at the lower edge of the pear-shaped hole to reduce surgical trauma and postoperative reactions.^49^ This appliance is suitable for Class III malocclusion with obvious maxillary underdevelopment. It is not suitable for patients with poor general condition, poor blood coagulation function, intolerance to implant surgery, and poor bone quality.^50^ The traction force on each side can be over 500 g, and it is worn for more than 13–16 h a day. Using a titanium plate-supported protraction reverse-pull headgear^51^ (Fig. 7) for maxillary growth modification treatment usually takes a year.

### Maxillary protraction with intermaxillary elastics to miniplates

**Fig. 8:**
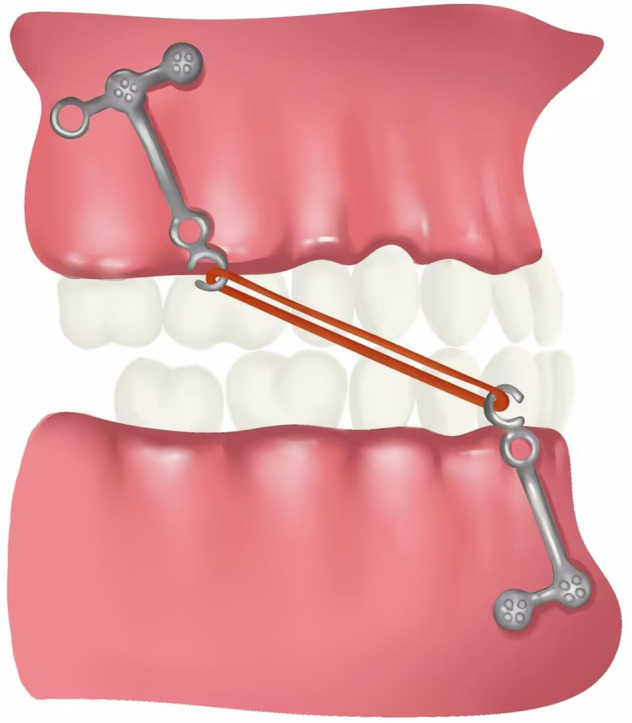
Schematic diagram of Maxillary protraction with intermaxillary elastics to miniplates

Compared with the traditional reverse-pull headgear, maxillary protraction with intermaxillary elastics to miniplates^52^ (Fig. 8) has the advantages of being worn all day and directly acting on the upper and lower jaws.^53^ Continuous intraoral forces give a better effect than intermittent extraoral traction.^54^ However, it requires surgery to place the titanium miniplates. Therefore, the contraindications for this therapy are similar to that of the bone-borne reverse-pull headgear. Generally, the traction starts 2–3 weeks after the titanium plates placement. The initial elastic force on each side is 150 g, which increases by 50 g each month until it reaches 250 g and is worn all day. Fixed orthodontic appliances can be used simultaneously for treatment. The general treatment course is 9–14 months.^55^

Bone-anchored maxillary protraction (BAMP) using Class III elastics attached to titanium miniplates, produced favorable skeletal effects in late-treatment groups. Recently, A hybrid Hyrax (HH) expander as anchorage in the maxilla and modified miniplates in the mandible to anchor Class III elastics in young patients (mean age, 10.6 years) was used. The study described a miniscrew anchored maxillary protraction (MAMP) protocol using an HH expander in the maxilla and two miniscrews in the mandible.^56^

### “2 × 4” orthodontic appliance

**Fig. 9:**
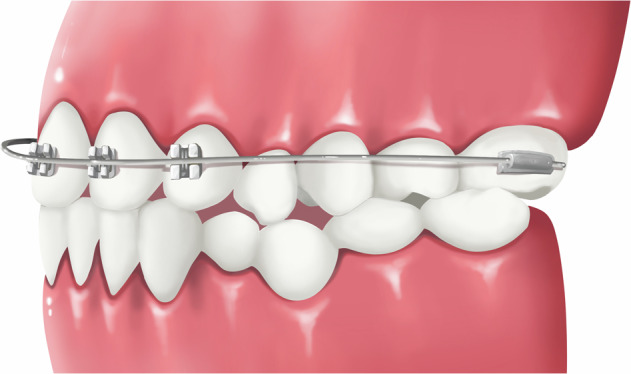
Schematic diagram of “2 × 4” orthodontic appliance

The correction of anterior crossbite is achieved by using the first permanent molar (or second deciduous molar) to provide anchorage.^57^ The “2 × 4” orthodontic technique (Fig. 9) is particularly suitable for mixed dentition patients with dental anterior crossbite, especially for those with normal or lingual-tipping upper incisors and labial-tipping lower incisors with spacing and rotated anterior teeth.^58^ This technique should be used with particular attention to using light force. It usually takes 6 months to use the “2 × 4” appliance to correct anterior crossbite.

### Conventional straight-wire appliance

**Fig. 10:**
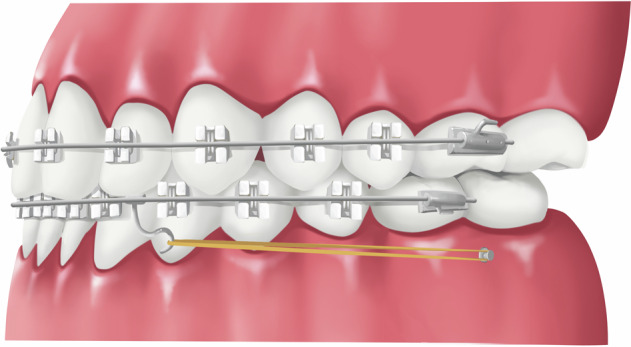
Schematic diagram of conventional straight-wire appliance

As a mature orthodontic technique, the straight-wire appliance (Fig. 10) can be used for comprehensive treatment of various malocclusions. For dental/functional and mild to moderate skeletal Class III malocclusions, the straight-wire appliances can be used to align the teeth, correct the anterior crossbite, and establish a neutral molar relationship, with or without extraction. For mild to moderate skeletal Class III malocclusions, temporary anchorage devices (TADs) can be used to facilitate the lower dentition distalization. For severe skeletal Class III malocclusions, combined orthodontic and orthognathic surgery is required.

### Transmission straight-wire appliance

**Fig. 11:**
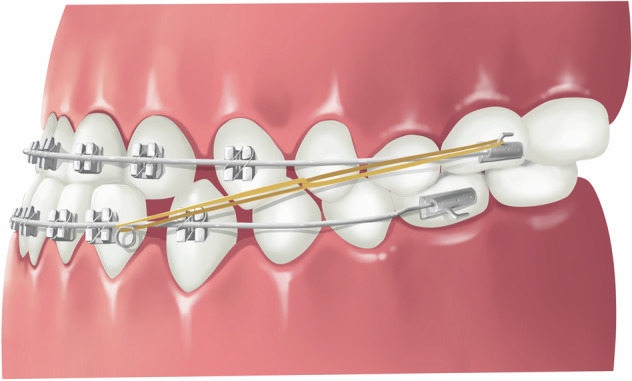
Schematic diagram of transmission straight-wire appliance

The “transmission straight-wire appliance” (Fig. 11) combines the advantages of both conventional straight-wire appliances and Begg appliances. In the early stage of treatment, fine wires and light force are used together with Class III elastics to quickly correct anterior crossbite, establish normal overbite and overjet, and enhance patients’ confidence in treatment. In the later stage of treatment, rectangular wires are used to express the pre-set data of the bracket to achieve precise teeth control. This technique opens up a new way for non-surgical treatment of skeletal Class III malocclusion.^59,60^ The “transmission straight-wire technique” emphasizes healthy light force correction, shortens the treatment duration, significantly reduces orthodontic complications such as root resorption, alveolar bone fenestration/dehiscence, and achieves healthy, efficient, precise and stable correction of Class III malocclusion.

### Clear aligners

**Fig. 12:**
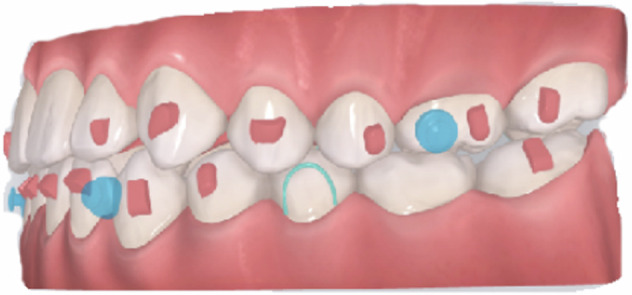
Schematic diagram of clear aligners

As a new orthodontic appliance characterized by 3D design and manufacture, the clear aligners (Fig. 12) can also be used for early orthodontic treatment of Class III malocclusion. This kind of appliance has a small impact on oral hygiene, eating, and pronunciation, with the advantages of good comfort and esthetic appearance. Class III intermaxillary elastics or TADs can be used to retract the lower anterior teeth, correct anterior crossbite, and adjust the molar relationship.

### Lingual orthodontic appliance

**Fig. 13:**
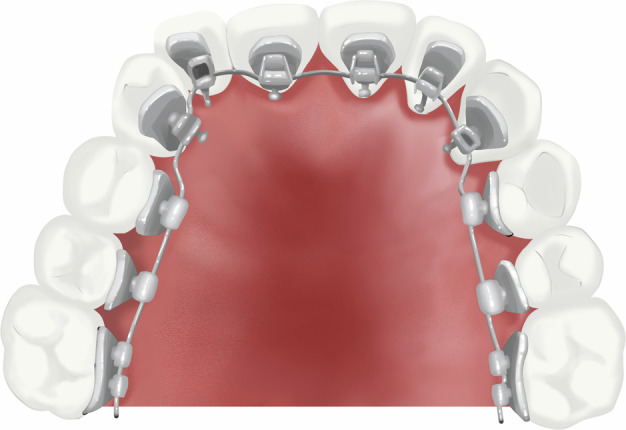
Schematic diagram of lingual orthodontic appliance

This technology provides a choice for patients with high esthetic requirements. Especially for adolescent patients, the technique avoids the demineralization of the labial surface of the teeth caused by acid etching during the placement of labial orthodontic appliances, thus protecting the labial surface of the teeth.^61^ Additionally, the unique bonding position of the brackets, which is closer to the center of resistance of the tooth, provides lingual orthodontics with advantages over labial orthodontics in terms of intruding anterior teeth, opening the bite, and maxillary expansion.^61,62^ However, lingual appliance (Fig. 13) is relatively expensive, and there is a higher possibility of bracket detachment occurring in adolescents, so the application of lingual appliance in adolescents is relatively limited.

### Comparative assessment

Current evidence on orthopedic treatment for Class III malocclusion demonstrates that orthopedic appliances can significantly improve jaw growth in growing patients with Class III malocclusion in the short-term. However, each appliance has certain disadvantages: reverse-pull headgear protrudes the maxilla, causing the lower jaw to rotate backward, which increases anterior facial height. Compared to dentally anchored devices, skeletally anchored devices can achieve greater horizontal maxillary movement with less tooth changes. Chin cup can retard mandibular growth.^63^ A single-center, prospective randomized controlled trial showed that for milder skeletal Class III malocclusion, reverse-pull headgear therapy combined with skeletal anchorage exhibits fewer side effects and provides orthopedic forces to the maxillary complex more efficiently than reverse-pull headgear therapy alone.^64^ This supports the advantages of skeletal anchorage with miniplates in treating patients with maxillary deficiency. Such an approach is particularly useful when patients have difficulty cooperating with reverse-pull headgear or when psychosocial factors affect the child’s treatment compliance. Moreover, skeletal anchorage treatment typically results in a shorter treatment time compared to extraoral devices. In comparison with miniplates, the use of mini-implants offers a lower cost, easier insertion, and greater patient comfort during the placement surgery. These treatments may also reduce the chances of having to undergo orthognathic surgery in adulthood.^65^ Reverse-pull headgear treatment allows obtaining more favorable effects in the control of mandibular position and a greater mandibular anterior morphogenetic rotation, making it a preferred choice for Class III patients with significant mandibular protrusion.^66^ The effect of maxillary protraction through reverse-pull headgear did not cause retraction of the mandibular incisors or significant protraction of the maxillary incisors.

## Early orthodontic treatment of different types of class III malocclusion (Table 2)

The timing of early orthodontic treatment for Class III malocclusion is very important. For anterior crossbite in the deciduous dentition period, the best time for treatment is around 3.5–5 years old, when the deciduous teeth roots have fully developed and not yet started to be absorbed, resulting in good treatment effect. If the treatment is too early, it may cause primary tooth instability and children may not cooperate well. If the treatment is delayed, the root of the deciduous incisors has already started to be absorbed, which makes it easy for the deciduous incisors to fall off when force is applied. Eliminating the crossbite condition of deciduous teeth can help guide the normal eruption of inherited permanent teeth and reduce the possibility of functional crossbite developing into skeletal crossbite due to occlusion interference. For crossbite in the mixed dentition period, treatment can be carried out when the permanent incisors’ roots have basically completed development, which is about 8–9 years old. If the treatment is carried out too early when the roots are not fully developed or if excessive orthodontic force is used, it may affect the development of permanent incisors and cause root absorption.^67^ Although some patients have undergone orthodontic treatment for crossbite during the primary dentition and mixed dentition period, with the onset of adolescence and the acceleration of growth and development, the anterior crossbite and sagittal discrepancy will once again appear or become significantly worse, often making the treatment more complicated. Therefore, for the treatment of Class III malocclusion, correct diagnosis and analysis of mechanism are crucial. For Class III malocclusion caused by excessive development of the mandible, the treatment should be appropriately delayed. Once the growth is mostly completed, the treatment plan should be determined based on whether combined orthodontic and orthognathic treatment or orthodontic camouflage treatment is required.

**Table 2:** Treatment approaches vary based on age and underlying etiology

Dentition	Dental	Functional	Skeletal
Primary Dentition	*Moderate or less severe reverse overbite: use Z-spring and bite plate to correct anterior crossbite *Deep reverse overbite or poor compliance: use lower inclined bite plane	*Eliminate occlusal interference or restore the posterior teeth *Correct bad habits *Use Z-spring and bite plate or lower inclined bite plane or Fränkel-III functional appliance to correct anterior crossbite	*Promote maxillary growth: use reverse-pull headgear and removable intra-oral appliance *Inhibit mandibular growth: use chin cup
Mixed Dentition	*Use removable appliance or 2 × 4 fixed appliance to correct anterior crossbite	*Eliminate occlusal interference or use functional appliance to guiding the mandible back to the normal position	*Maxillary hypoplasia: use maxillary protraction *Excessive mandibular growth: use chin cup
Permanent Dentition	*Mild to moderate dental crowding: non-extraction, labial inclination of upper incisors or maxillary expansion appliances to gain space *Severe crowding: extraction of premolars	*Use functional appliance for first-stage treatment *Use fixed orthodontic appliance or clear aligners for second-stage treatment	*Remaining growth potential: a combination of maxillary palatal expansion and forward protraction *Significant overgrowth of the mandible and a tendency for asymmetry: delay treatment until the end of growth peak *Mild to moderate skeletal Class III malocclusions: camouflage techniques *Severe skeletal deformities: surgical treatment

### Early orthodontic treatment of dental class III malocclusion

Due to the obstacles in the process of tooth eruption and replacement, the abnormal position of the maxillary and mandibular incisors leads to simple anterior crossbite. This kind of anterior crossbite usually has neutral or slightly mesial molar relationship (the mandibular first molar moves proximally within 1/2 cusp width), with the maxillary anterior teeth being lingually inclined or normal, and the mandibular anterior teeth being labially inclined or normal; skeletal Class I (0° ≤ ANB angle ≤ 5°, Wits value between 2.0 and −2.0 mm), and the vertical facial height is within the normal range or slightly reduced.^68–70^ Treatment is generally easier and has a good prognosis.^71,72^ The treatment is aimed at alveolar problems, eliminating anterior teeth crossbite, expanding the upper arch and establishing Class I molar relationship.

#### Primary dentition stage

It is often manifested as the maxillary incisors being lingually tipping or normal, and the mandibular incisors being labially inclined with spacing. The mid-primary dentition stage (around 4 years old) is a good time for orthodontic intervention. Patients with moderate or less severe reverse overbite can use the removable appliance incorporating z-spring and posterior bite plate;^73^ patients with deep reverse overbite or poor compliance can use a lower inclined bite plane. In the late primary dentition stage (5–6 years old), due to the maxillary and mandibular deciduous incisors being close to replacement and often loose, it is not recommended to directly apply orthodontic force to the upper and lower deciduous anterior teeth, which may cause the deciduous teeth to fall off earlier.

#### Mixed dentition stage

For dental crossbite, it can be corrected by labial proclination of the upper anterior teeth and lingual retraction of the lower anterior teeth. Commonly used appliances include “removable appliance incorporating Z-spring and posterior bite plate” and 2 × 4 fixed appliance. For patients with anterior teeth rotation or spacing, 2 × 4 fixed appliance can be used to correct the anterior crossbite while in the meantime solve other problems.

#### Early permanent dentition stage

The correction of dental anterior crossbite is part of comprehensive orthodontic treatment. Whether it is necessary to extract teeth for correction depends on the degree of dental crowding. For some patients with mild to moderate dental crowding, jaw deformity and compensation of teeth, extraction is generally not required. Labial inclination of upper incisors can provide extra space for alignment. Therefore, for patients with mild crowding of the upper dentition and relatively upright or even lingually inclined upper incisors before treatment, non-extraction correction can be applied. Maxillary expansion appliances can also be used to gain space for alignment and crossbite correction. For patients with more crowding in upper dentition or obviously proclined lower incisors, extraction of premolars could provide more space for differential retraction of the upper and lower anterior teeth to establish normal overjet and adjustment of the molar relationship to Class I.^71^ The decision for extraction or non-extraction is mainly determined by the upper dentition, considering factors such as the degree of crowding, width of the maxillary arch, and the labial inclination of the upper incisors.^74,75^

### Common extraction patterns^75^

#### Extraction of four first premolars

This pattern can be chosen when there is severe crowding or protrusion of the anterior segment of the maxillary and mandibular dental arches. The typical clinical manifestations include anterior crossbite, severe upper dentition crowding, slightly mesial molar relationship, labial inclination of the lower anterior teeth and mild skeletal deformity.

#### Extraction of upper second and lower first premolars

This is the most common extraction pattern in Class III malocclusion camouflage treatment. It is recommended in cases with anterior crossbite, obvious mesial molar relationship, moderate crowding in upper dentition, lower dentition with proclined lower incisors and mild skeletal deformity.

### Early correction of functional class III malocclusion

Functional anterior crossbite and mandibular protrusion, which often caused by occlusal interference, early contact and bad oral habits, are primarily classified as functional Class III malocclusion. Functional anterior crossbite is usually associated with mild mesial molar relationships, a generally normal size and shape of the mandible but with a forward position, showing a mild mandibular protrusion and a Class III skeletal profile. The typical feature of functional anterior crossbite is that the mandible can recede to the position where the upper and lower anterior teeth are edge to edge relation. When the mandible is in a retruded position or postural position, the ANB angle significantly increases, and the profile also improves significantly compared to the occlusal position.^76^ The treatment response of simple functional anterior crossbite is good, with a favorable prognosis. The correction of functional Class III malocclusion generally involves changing the position of the mandible to achieve consistency and coordination between tooth and muscle positions, thereby resolving the anterior crossbite.^75^

#### Primary dentition stage

As occlusal interference and early contact are the main reasons that induce functional Class III malocclusion, it is necessary to eliminate these factors and correct bad habits first. Adjustment grinding can be used for correction, which involves grinding the lingual side of the incisal edge of the mandibular incisors and the labial side of the incisal edge of the maxillary incisors to release the anterior teeth from the crossbite status. Special attention should be paid to modifying unworn primary canines so that there is no occlusal interference when the mandible closes, allowing it to return to the normal position. If the crossbite is caused by habitual forward positioning of the mandible due to posterior tooth decay and loss, the caries should be treated, and temporary restoration should be made for the missing posterior teeth to restore posterior chewing. At the same time, training should be provided for children to overcome the habit of protruding the mandible. A removable appliance with Z-spring and posterior bite plate or lower inclined bite plane can be used for correction of anterior crossbite. The best time for correction in primary dentition is around 3.5–5 years old, with a treatment duration of 3 to 6 months. Functional Class III malocclusion can also be treated by wearing a Fränkel-III functional appliance, and a treatment duration of about one year.

#### Mixed dentition stage

This is a critical period for treating anterior crossbite.^77^ For functional crossbite, the primary goal is to eliminate functional factors, such as removal of occlusal interference, alignment of the palatally malpositioned lateral incisors and intruding lower anterior teeth to reduce the reverse deep bite and guiding the mandible back to the normal position. The Fränkel-III functional appliance and 2 x 4 appliance can achieve good results.^78^

#### Early permanent dentition stage

The anterior crossbite at this stage may be a combination of functional and skeletal factors. Therefore, it is necessary to distinguish the type of existing malocclusion and predict the development trend of the crossbite. For functional anterior crossbite, a removable appliance such as Fränkel-III can be used first to resolve the anterior crossbite, followed by a second-stage treatment.^78^ The second-stage treatment can be performed using fixed orthodontic appliance or clear aligners, among which the “transmission straight wire appliance” has its unique advantage in treating Class III malocclusion both effectively and efficiently. For patients with mild to moderate crowding, non-extraction orthodontic treatment can be attempted. For patients with severe crowding, extraction orthodontic treatment may be chosen.

### Early correction of skeletal Class III malocclusion

Skeletal Class III malocclusion, also known as true Class III malocclusion, is characterized by abnormal intermaxillary relationships due to unbalanced growth of the maxilla and mandible, accompanied by significant dental compensation (the upper incisors are labially inclined, and the lower incisors are lingually inclined). The skeletal discrepancy (ANB angle < 0°) shows true skeletal problems without functional anterior shift of the mandible. Skeletal Class III malocclusion is one of the most challenging malocclusions to treat and may require orthognathic surgery for severe cases.^79^

#### Primary dentition stage

For cases showing deficient maxillary growth, a removable intra-oral appliance along with reverse-pull headgear can be used to promote maxillary growth and correct the anterior crossbite. However, for cases primarily showing a tendency for excessive mandibular growth, although chin cup can be attempted, its effect on inhibiting mandibular growth is very limited. The treatment of Class III malocclusion in the primary dentition stage is long-term, recurrent, and complex, and parents should be informed of this in advance.

#### Mixed dentition stage

For skeletal crossbites, it is necessary to determine if the problem lies in the maxilla or mandible. For maxillary hypoplasia, protraction is commonly used, and rapid palatal expansion before protraction can enhance the effectiveness. A functional appliance can be used during the observation period to maintain stability. For cases with excessive mandibular growth, treatment can be very challenging as it is difficult to restrict forward growth of the mandible. In such cases, the correction of crossbite mainly relies on compensation by the upper and lower anterior teeth, and the maxilla may need to be protracted slightly more if necessary.^80^

#### Early permanent dentition stage

For cases with remaining growth potential, a combination of maxillary palatal expansion and forward protraction can be attempted to promote maxillary growth. Tooth-supported or bone-supported reverse-pull headgears as well as miniplates-supported intermaxillary elastics can be used. For patients with significant overgrowth of the mandible and a tendency for asymmetry, it is suggested to observe and delay treatment until the end of growth peak.^81^ Then, based on the severity of skeletal deformity and the patient’s requirement for facial esthetic improvement, a decision need to be made regarding whether to accept combined orthodontic-orthognathic treatment or orthodontic camouflage treatment.^82^ It is essential to develop a definitive treatment plan for patients with skeletal Class III malocclusion at this stage. Mild to moderate skeletal Class III malocclusions can be treated with camouflage techniques, involving appropriate labial movement of upper anterior teeth and lingual movement of lower anterior teeth to correct anterior crossbites. For severe skeletal Class III malocclusion with significant dental compensation, surgical treatment should be first considered.

##### Retrognathic maxilla and normal mandible type

This is the most common type of skeletal Class III malocclusion.^30^ Typically, there is insufficient development of the midface region. The molars show Class III relationship, with the first mandibular molar moving mesially between half to a full cusp (4-6 mm). It is usually accompanied by both posterior and anterior crossbites. Treatment primarily focuses on correcting maxillary retrusion and crossbites. According to most published studies, treatment should start before age 10, with rapid maxillary expansion and reverse-pull headgear being commonly used methods.^43,83–93^ After expansion and protraction treatment, palatal bars can be used to maintain the width of the first molars, followed by comprehensive orthodontic treatment using fixed appliances or clear aligners. After treatment, it is recommended to extract the lower wisdom teeth and continue monitoring until the age of 18.

##### Normal maxilla and prognathic mandible type

This type is caused by excessive development of the mandible or forward displacement of the mandible, leading to an abnormal sagittal relationship between the maxilla and mandible. The molar relationship is usually moderate to severe Class III, showing complete mesial positioning of the mandible, especially in patients with excessive vertical development (high angle) and significant mandibular deviation. The anterior crossbite is usually associated with anterior open bite and lingually inclined lower incisors. These patients are more likely to require orthognathic surgery. It is recommended to observe at least until 16-17 years old before starting pre-surgical orthodontic treatment. For patients who do not accept orthognathic surgery, camouflage orthodontic treatment using “transmission straight-wire therapy” can be attempted.

##### Retrognathic maxilla with prognathic mandible type

This type of malocclusion is often characterized by underdevelopment of the mid-face and mandibular protrusion. The molar relationship is usually severe Class III, indicating more severe skeletal discrepancy. It is typically accompanied by both anterior and posterior crossbites. Compared to patients with Class I malocclusion, the growth rate of the mandible in patients with Class III malocclusion is faster during puberty. When this type of skeletal Class III is detected in the primary dentition or mixed dentition stage, the reverse-pull headgear therapy should be started as soon as possible to promote the normal development of maxilla and mandible. If not treated in time, these cases may develop into more severe skeletal Class III malocclusion, which limits treatment options and increases the likelihood of requiring combined orthodontic-orthognathic surgery in the later stage. For this type, the selection of tooth extraction should be cautious, and the possibility of combined orthodontic-orthognathic surgery should be fully considered.

##### Skeletal Class III malocclusion caused by craniofacial deformities

Craniofacial syndromes such as Crouzon syndrome, Apert syndrome, Pfeiffer syndrome, Saethree-Chotzen syndrome, Antley-Bixler syndrome, and Carpenter syndrome^94–96^ typically involve maxillary retrusion, severe skeletal open bite and Class III malocclusion. Early identification of these conditions is beneficial for early intervention during growth and development. For skeletal Class III malocclusions caused by various craniofacial syndromes, combined orthodontic-orthognathic treatment is often the preferred treatment option.

## Issues to be addressed during early orthodontic treatment of class III malocclusion

### Differential diagnosis of Class III malocclusion in terms of skeletal and functional problems

The differential diagnosis was mainly based on the presence of family history, mandibular functional shift, dental compensation of the anterior teeth and cephalometric analysis measurements. Skeletal Class III malocclusion generally does not have or only has a small amount of functional shift of the mandible, and the mandible cannot be moved backwards to the position where the upper and lower anterior teeth are in opposition. The proportion of Class III malocclusion in the patient’s immediate family members is relatively high. The upper anterior teeth are labially inclined, while the lower anterior teeth are lingually inclined. It is often a case of high angle and may have a tendency for anterior open bite.

### Selection of orthodontic treatment timing

For early mixed dentition, orthodontic treatment can be applied to dental, functional, mild-to-moderate skeletal Class III malocclusion. However, for severe skeletal Class III malocclusion, it is necessary to wait until the end of the growth spurt before deciding whether orthognathic surgery is necessary.

### Determination of primary and secondary orthodontic goals

The objective of phase I treatment for Class III malocclusion is to remove the influence of occlusal interference on growth and development and reduce the degree of skeletal deformity. The objective of phase II treatment is to align the dentition, improve facial profile, and establish a healthy and stable occlusion.

### Choice of appliance

Removable and functional appliances are mainly used for the treatment of primary and mixed dentition. For early permanent dentition, fixed appliances and clear aligners can be chosen. Among them, the “transmission straight-wire therapy” can be the preferred appliance for treating mild-to-moderate skeletal Class III malocclusion.

### Parents’ and patients’ cooperation

The treatment of Class III malocclusion has long-term, complex and recurrent characteristics. Regardless of the type of appliance used, patients need to strictly follow the doctor’s instructions and wear it for a sufficient period of time. Therefore, it is essential for parents to understand the importance of cooperation in the treatment of Class III malocclusion and fulfill their supervisory responsibilities to achieve good treatment results.

## Conclusions and expectations

The causes of Class III malocclusion are diverse, and early prevention is necessary. It is essential to timely remove systemic and local adverse factors that affect the normal growth and development of teeth (including primary and permanent teeth), alveolar bone and jaws, so that the normal occlusal relationship of dental arches can be established, maxilla and mandible can develop normally, facial growth can be coordinated and sound functions of the maxillofacial system can be achieved.

The timing of early orthodontic treatment for malocclusion is crucial and should be determined through comprehensive assessment of chronological age, dental age, and skeletal age. Early prevention and treatment of Class III malocclusion should be promoted through various channels to educate parents and children about basic knowledge on preventing dental and jaw malformations. Through the cooperation of orthodontists, patients and parents, we can work together to promote oral health care for children and early prevention and treatment of dental and jaw malformations. As the complexities of early orthodontic intervention to Class III malocclusion. The unpredictable nature of the malocclusion’s progression and the potential variability in treatment outcomes should be fully taken into account in early treatment of Class III malocclusion. Early intervention strategies should not be overused clinically.
